# Translational control mechanism of HIV-1 *tat1* mRNA

**DOI:** 10.1186/1742-4690-8-S2-P39

**Published:** 2011-10-03

**Authors:** Georges Khoury, Lilia Ayadi, Christiane Branlant

**Affiliations:** 1 AREMS, UMR 7214 CNRS-UHP, Biopôle, faculté de médecine, 54506 Vandoeuvre-lès-Nancy, France

## Background

The Human Immunodeficiency Virus type 1 Tat protein is a major viral transactivator which stimulates the synthesis of full length transcripts by interacting with the 5'end of all nascent viral RNAs. Translation of *tat* mRNAscan be cap dependent. Nevertheless, in phaseG2/M ribosomes translate *tat* mRNAs by an alternative initiation processdepending on anlnternal Ribosome Entry Site (IRES). This initiation is auto-stimulated by Tat [1]. Recently, SRp40 and SRp55 proteins were found to promote Gag IRES-dependent translation initiation [2]. Therefore, SR and hnRNP proteins likely play a key role in regulation of both splicing and translation of HIV-1 RNA. As we previously observed a binding of hnRNP H (3), DAZAP1 and various SR proteins in the vicinity of the *tat* initiation codon(4), we asked whether these proteins can regulate *tat* mRNA translation.

## Materials and methods

To address this issue, we built mono and bicistronic constructs expressing a Tat-Renilla luciferase fusion protein or individual Tat and Renilla proteins, respectively. We used these constructs to studythe effect of an over-expression of SR and hnRNPproteins in Hela cells on the cap dependent and cap-independent*tat1* mRNA translation initiation. Efficiency of Tat production by the bi-cistronic construct was tested by measuring the Tat activity on a Firefly luciferase gene under the control of the HIV-1 LTR.

## Results

Our data strongly suggest that hnRNP H, and some of the SR proteins (ASF-SF2 and 9G8)activate the *tat1* mRNA IRES, whileDAZAP1 inhibit its activity, and these regulationsdepends uponthe presence of the *tat1* mRNA5'UTR.

## Conclusions

Altogether our data reveal a complex regulation of the internal initiation of *tat1* mRNA, that may reflect the situation in human cells infected by virus HIV-1.
